# Clinical outcome of biodegradable polymer sirolimus-eluting stent and durable polymer everolimus-eluting stent in patients with diabetes

**DOI:** 10.1186/s12933-020-01145-x

**Published:** 2020-10-01

**Authors:** Ryota Kakizaki, Yoshiyasu Minami, Masahiro Katamine, Aritomo Katsura, Yusuke Muramatsu, Takuya Hashimoto, Kentaro Meguro, Takao Shimohama, Junya Ako

**Affiliations:** Department of Cardiovascular Medicine, Kitasato University School of Medicine, 1-15-1 Kitasato, Minami-ku, Sagamihara, Kanagawa 252-0373 Japan

**Keywords:** Drug-eluting stent, Percutaneous coronary intervention, Target lesion revascularization

## Abstract

**Background:**

Diabetes mellitus is a risk for increased incidence of adverse clinical events after percutaneous coronary intervention. However, the difference in the incidence of adverse clinical events according to stent type in patients with diabetes remains to be elucidated. In the present study, we aimed to compare the clinical outcomes between patients treated with the biodegradable polymer sirolimus-eluting stents (BP-SES) and the durable polymer everolimus-eluting stents (DP-EES) among patients with diabetes.

**Methods:**

Among 631 lesions in 510 consecutive patients treated with either BP-SES or DP-EES, 165 lesions in 141 patients with diabetes mellitus and stable angina pectoris were identified and classified into the BP-SES group (48 lesions in 44 patients) and the DP-EES group (117 lesions in 100 patients). The incidence of adverse clinical events after stent implantation was compared between the 2 groups.

**Results:**

There was no significant difference in the prevalence of conventional risk factors, lesion characteristics, and procedural characteristics between the 2 groups. During median 386 [334–472] days follow-up, the incidence of target lesion revascularization (11.4 vs. 2.0%, *p* = 0.003) and device-oriented clinical endpoint (13.6 vs. 6.0%, *p* = 0.035) in the BP-SES group was significantly greater than that in the DP-EES group. A univariate model demonstrated that the BP-SES usage was significantly associated with the higher incidence of target lesion revascularization (odds ratio, 6.686; 95% confidence interval, 1.234–36.217; *p* = 0.028).

**Conclusion:**

BP-SES was associated with the greater incidence of TLR than the DP-EES in patients with diabetes mellitus. Further studies with larger cohorts and longer follow-up are required to confirm the present results.

## Background

Performance of coronary stent has been improved to reduce the incidence of adverse events including stent thrombosis and repeat revascularization [1]. Although the bare metal stents (BMS) was developed to overcome the limited efficacy of plain old balloon angioplasty, the need for repeat revascularization caused by neointimal hyperplasia was still a problem [2]. Drug-eluting stents (DES) was designed to suppress excessive neointimal growth by anti-proliferative drug released from polymer around stent strut. In fact, DES significantly reduced the incidence of repeat revascularization compared with BMS [3]. However, the earlier generation DES still had several concerns for the incidence of adverse events including late stent thrombosis, which might be caused by DES components [4]. Thus, continuous efforts have been made to develop newer DES with biocompatible drug, polymer and metal [5]. The biodegradable polymer sirolimus-eluting Ultimaster™ stent (BP-SES) (Terumo, Tokyo, Japan) is a new-generation sirolimus-eluting stent consisting of a thin strut cobalt-chromium platform with an abluminal gradient coating of sirolimus-releasing biodegradable polymer that is completely resorbed within 3–4 months [6]. Several studies showed comparable clinical outcomes between patients treated with the BP-SES and those treated with durable polymer 2nd-generation DES. The CENTURY II trial demonstrated the noninferiority in the incidence of target lesion failure (TLF) at 9 months in patients treated with the BP-SES compared with those treated with the durable polymer everolimus-eluting stent (DP-EES) [7]. In the ULISSE registry, which includes 1660 patients in a real-world cohort in Italy, the incidence of TLF within 1 year after BP-SES implantation was reported as 5% [8], which was numerically comparable to that after 2nd-generation DES implantation in previous studies [9, 10].

The higher incidence of adverse events in patients with diabetes than those without diabetes is an unsolved problem of percutaneous coronary intervention. It has been demonstrated after the implantation of bare metal stents [11], first-generation drug-eluting stents [12], second- and third-generation DES [13–15] including Ultimaster™ BP-SES [16]. The difference in the incidence of adverse events in patients with diabetes among the type of DES has been also investigated. Several previous studies demonstrated the comparable clinical outcomes in some biodegradable polymer sirolimus-eluting stents and second-generation DESs including DP-EES in patients with diabetes [17–19]. However, the difference in the incidence of adverse clinical events between patients with Ultimaster™ BP-SES and those with other DESs among patients with diabetes has not been fully evaluated, although the Ultimaster™ BP-SES has a unique structure such as abluminal polymer with gradation coating within the central part of strut [6, 20]. In the present study, we aimed to compare the clinical outcomes between patients treated with the BP-SES and those treated with the DP-EES in patients with diabetes.

## Methods

### Study population

**Fig. 1:**
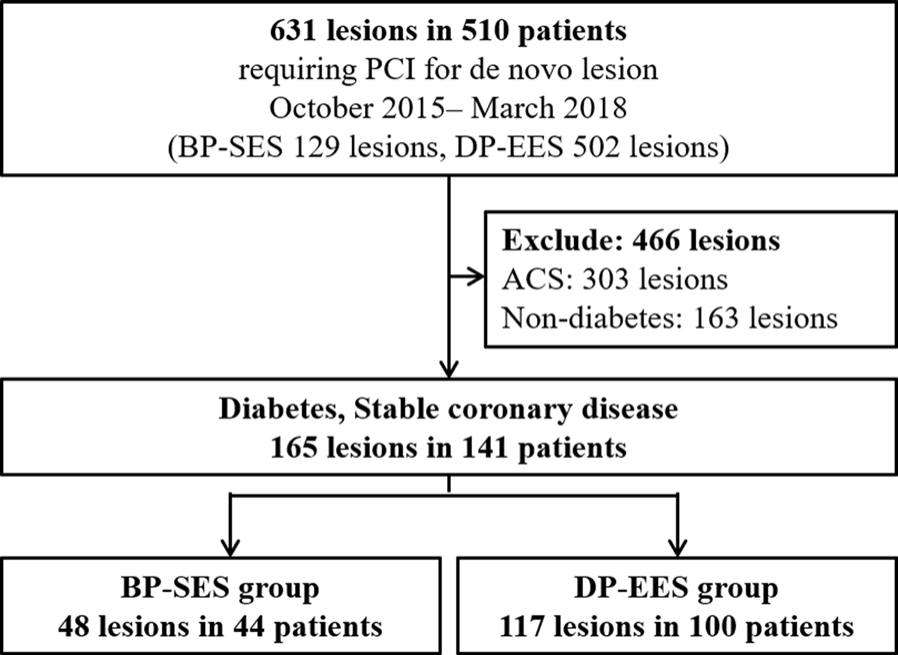
Study flow chart. ACS, acute coronary syndrome; BP-SES, biodegradable polymer sirolimus-eluting stent; *DM* diabetes mellitus, *DP-EES* durable polymer everolimus-eluting stent, *PCI* percutaneous coronary intervention

This was a single-center, retrospective, observational study. A total of 631 consecutive lesions in 510 patients underwent percutaneous coronary intervention (PCI) with either BP-SES (Ultimaster™ sirolimus-eluting stent, TERUMO, Tokyo, Japan) or DP-EES (Xience™ everolimus-eluting stent, Abbott, Santa Clara, CA, USA) between October 2015 and March 2018 were enrolled. Among them, 165 lesions in 141 patients with diabetes mellitus and stable angina pectoris were identified and classified into the DP-SES group (48 lesions in 44 patients) and the DP-EES group (117 lesions in 100 patients) (Fig. 1). Because there might exist potential bias in baseline clinical characteristics between the 2 groups, we further compared the incidence of adverse events in an adjusted cohort, which was identified using a propensity score-matched analysis (Additional file 1). The study protocol complied with the principles of the Declaration of Helsinki and was approved by the Human Research Committee of Kitasato University School of Medicine. All patients provided written informed consent before the procedure.

### Percutaneous coronary intervention

PCI was performed in accordance with recommended standard strategies although all procedural methods including stent selection were decided by the operators. There was no significant time trend in using BP-SES and DP-EES in our institute (Additional file 1: Table S1). Before the stent implantation, all patients received aspirin 100 mg and clopidogrel 75 mg or prasugrel 3.75 mg from at least 5 days before the day of stent implantation. Unfractionated heparin (5000 IU, bolus) was administered just before percutaneous coronary intervention. In addition to bolus injection of heparin, additional unfractionated heparin was administered to maintain an activated clotting time of > 250 s during the procedure. Dual antiplatelet therapy was maintained for at least 6 months unless the patient had serious adverse effects or a surgical procedure.

### Study endpoint and definitions

Clinical follow-up data were obtained from the medical records of the outpatient clinic. The primary outcome measure was ischemia-driven target lesion revascularization (TLR). The secondary outcome measure was device-oriented clinical endpoint (DoCE), which was recorded cumulatively and hierarchically, including cardiac death, target vessel-related myocardial infarction, TLR, and stent thrombosis. More than 1 event recorded in the same patient at the same time point was attributed as 1 composite cardiac event for statistical analysis. Major adverse cardiac event was defined as the composite of cardiac death, myocardial infarction, stent thrombosis, and TLR. Further details regarding the study endpoint and definition are described in Additional file 1.

### Cost-effectiveness analysis

The cost-effectiveness of the 2 stents was analyzed. The cumulative cost of initial procedure, following standard care and cardiac events within 1 year (Additional file 1: Figure S1) was calculated in each group. The cost per patient within 1 year was compared between the 2 stents. We used quality-adjusted life-year (QALY) as an outcome measurement in analysis for incremental cost-effectiveness ratio (ICER) [21]. Further details are described in Additional file 1.

### Statistical analysis

Continuous outcome data were summarized as mean ± standard deviation, whereas the median value with interquartile range was reported when data were not normally distributed. The comparison of continuous variables was undertaken using t-test or Mann–Whitney U test. Categorical outcome data were summarized as counts (percentage). Between-group comparisons were performed using Fisher’s exact test or the chi-squared test, as appropriate, depending on the expected frequency distribution under the null hypothesis. Further details are described in Additional file 1. The comparisons of cumulative incidences of adverse events between the 2 groups were conducted using Fine-Gray model to adjust competing risks. The Generalized Estimating Equations approach was used to take into account the within-subject correlation due to multiple lesions analyzed within a single patient. Logistic regression analyses were performed to determine the factors for the incidence of each clinical events. The variables with *p* < 0.05 in univariate analysis were included in the multivariate model. Statistical significance was defined as *p* < 0.05. All statistical analyses were performed with SPSS 24.0 (SPSS Inc., Chicago, IL), JMP 13.2.1 (SAS Inc., Cary, NC), and R version 4.0.2. (R Foundation for Statistical Computing, Vienna, Austria.).

## Results

### Baseline characteristics

Baseline clinical characteristics are shown in Table 1. There were no significant differences in baseline clinical characteristics between the 2 groups other than the rate of thienopyridine and beta-blocker administration. Patients requiring hemodialysis was 14.9%. There was no significant difference in duration of dual antiplatelet therapy between the 2 groups [301 (108–447) vs. 322 (123–398) days, *p* = 0.777]. Lesion and procedural characteristics are shown in Table 2. There were no significant differences in lesion and procedural characteristics between the 2 groups. Baseline clinical characteristics, lesion and procedural characteristics in an adjusted cohort are shown in Additional file 1: Tables S2, S3.

**Table 1:** Clinical characteristics

	Overall	BP-SES	DP-EES	*p* value
	n = 141	n = 44	n = 100	
Age, years	70 (62–77)	67 (57–78)	70 (64–77)	0.278
Male, n (%)	115 (81.6)	32 (72.7)	85 (85.0)	0.082
Body mass index	24.7 (22.0–27.1)	24.8 (22.4–26.7)	23.8 (22.4–26.7)	0.856
Follow-up duration, days	386 (334–472)	377 (308–435)	390 (347–484)	0.139
Risk factor, n (%)				
Hypertension	122 (86.5)	40 (90.9)	85 (85.0)	0.335
Dyslipidemia	109 (77.3)	35 (79.6)	76 (76.0)	0.641
Chronic kidney disease	87 (61.7)	25 (56.8)	64 (64.0)	0.414
Hemodialysis	21 (14.9)	6 (13.6)	15 (15.0)	0.831
Smoking	25 (17.9)	10 (22.7)	15 (15.2)	0.271
Family history of IHD	36 (26.1)	14 (32.6)	23 (23.5)	0.259
Previous myocardial infarction	42 (29.8)	9 (20.5)	35 (35.0)	0.081
Previous PCI	59 (41.9)	15 (34.1)	47 (47.0)	0.150
Previous CABG	5 (3.5)	3 (6.8)	2 (2.0)	0.146
Medication at PCI, n (%)				
Aspirin	125 (88.7)	37 (84.1)	90 (90.0)	0.311
Thienopyridine	114 (80.9)	31 (70.5)	86 (86.0)	*0.028*
Statin	116 (82.3)	36 (81.8)	83 (83.0)	0.863
ACEI/ARB	109 (77.3)	30 (68.2)	81 (81.0)	0.092
Beta blocker	93 (66.0)	22 (50.0)	74 (74.0)	*0.005*
Calcium channel blocker	70 (49.6)	19 (43.2)	51 (51.0)	0.387
Insulin	26 (18.6)	6 (14.0)	21 (21.0)	0.324
Laboratory findings				
Triglycerides, mg/dL	125 (92–185)	116 (91–176)	125 (92–197)	0.284
LDL-C, mg/dL	85 (67–109)	90 (68–107)	80 (66–112)	0.700
HDL-C, mg/dL	49 (41–58)	51 (42–66)	48 (40–57)	0.112
HbA1c, %	6.8 (6.3–7.3)	6.8 (6.3–7.4)	6.8 (6.3–7.3)	0.907
eGFR, mL/min/1.73 m^2^	52 (34–64)	55 (35–67)	51 (32–63)	0.253
BNP, pg/mL	108 (46–322)	79 (27–299)	115 (54–350)	0.108
Left ventricular ejection fraction, %	60 (50–65)	61 (51–66)	59 (50–65)	0.293

*ACEI* angiotensin converting enzyme inhibitor, *ARB* angiotensin II receptor blocker, *BNP* brain natriuretic peptide, *BP-SES* biodegradable polymer sirolimus-eluting stent, *CABG* coronary artery bypass graft, *DP-EES* durable polymer everolimus-eluting stent, *eGFR* estimated glomerular filtration rate, *HbA1c* hemoglobin A1c, *HDL-C* high density lipoprotein cholesterol, *IHD* ischemic heart disease, *LDL-C* low-density lipoprotein cholesterol, *PCI* percutaneous coronary intervention

**Table 2:** Lesion and procedural characteristics

	Overall	BP-SES	DP-EES	*p* value
	n = 165	n = 48	n = 117	
Target vessel, n (%)				0.254
Left main trunk	7 (4.2)	1 (2.1)	6 (5.1)	
Left anterior descending artery	86 (52.1)	25 (52.1)	61 (52.1)	
Left circumflex artery	20 (12.1)	3 (6.2)	17 (14.5)	
Right coronary artery	52 (31.6)	19 (39.6)	33 (28.3)	
Type B2/C lesion, n (%)	92 (55.8)	30 (62.5)	62 (53.0)	0.264
QCA pre PCI				
Lesion length, mm	22.6 (12.7–35.1)	21.3 (12.2–37.9)	23.9 (13.5–35.0)	0.401
Reference vessel diameter, mm	2.48 (2.07–2.98)	2.45 (2.11–2.81)	2.48 (2.06–3.02)	0.649
Minimum lumen diameter, mm	0.87 (0.45–1.22)	0.78 (0.51–1.01)	0.91 (0.42–1.30)	0.410
Diameter stenosis, %	64 (51–80)	69 (58–80)	63 (50–80)	0.405
QCA post PCI				
Reference vessel diameter, mm	2.63 (2.34–3.02)	2.67 (2.43–3.10)	2.60 (2.26–3.01)	0.321
Minimum lumen diameter, mm	2.30 (2.02–2.60)	2.39 (2.05–2.75)	2.26 (2.01–2.57)	0.216
Diameter stenosis, %	11 (5–18)	11 (5–17)	12 (5–18)	0.624
Rotational atherectomy, n (%)	12 (7.3)	2 (4.2)	10 (8.6)	0.325
Bifurcation, n (%)	38 (23.0)	13 (27.1)	25 (21.4)	0.428
Chronic total occlusion, n (%)	17 (10.3)	4 (8.3)	13 (11.1)	0.594
Imaging device, n (%)	165 (100)	48 (100)	117 (100)	–
Number of stents, n	1 (1–2)	2 (1–2)	1 (1–2)	0.100
Total stent length, mm	33 (23–56)	37 (27–62)	33 (21–54)	0.134
Minimum stent diameter, mm	2.75 (2.5–3.0)	2.5 (2.5–3.0)	2.75 (2.5–3.0)	0.468

*BP-SES* biodegradable polymer sirolimus-eluting stent, *DP-EES* durable polymer everolimus-eluting stent, *PCI* percutaneous coronary intervention, *QCA* quantitative coronary angiography

### Clinical outcomes

**Fig. 2:**
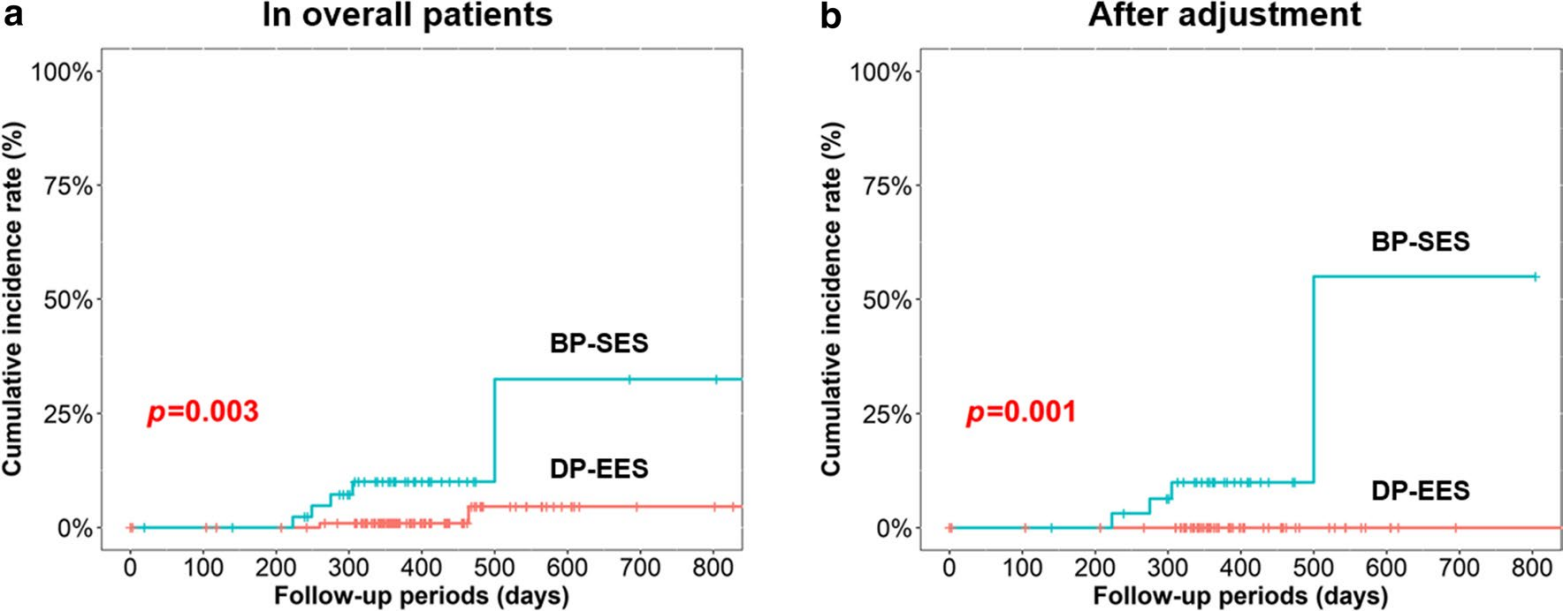
Target lesion revascularization in the 2 stents. **a** Overall cohort; **b** Adjusted cohort; *BP-SES* biodegradable polymer sirolimus-eluting stent, *DP-EES* durable polymer everolimus-eluting stent

**Fig. 3:**
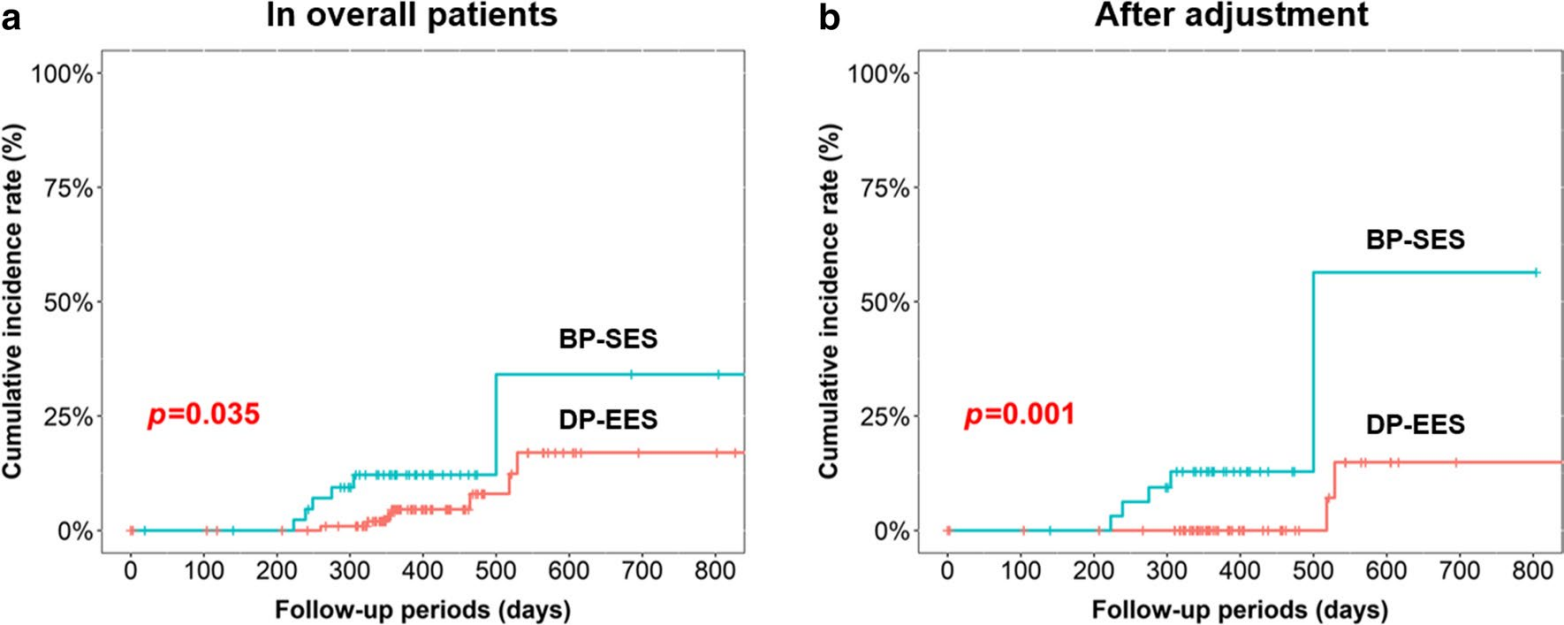
Device-oriented clinical endpoint in the 2 stents. **a** Overall cohort; **b** adjusted cohort; *BP-SES* biodegradable polymer sirolimus-eluting stent, *DP-EES* durable polymer everolimus-eluting stent

The median follow-up duration was 386 (334–472) days. The cumulative incidence of TLR was significantly lower in the BP-SES group than in the DP-EES group in both of overall cohort and an adjusted cohort (11.4 vs. 2.0%, *p* = 0.003, 12.9 vs. 0%, *p* = 0.001, respectively) (Fig. 2). The cumulative incidence of from DoCE was also significantly lower in the BP-SES group than in the DP-EES group in both of overall cohort and an adjusted cohort (13.6 vs. 6.0%, *p* = 0.035, 16.1 vs. 3.3%, *p* = 0.001, respectively) (Fig. 3). The details of adverse clinical events in an overall cohort and an adjusted cohort are summarized in Table 3 and Additional file 1: Table S4.

**Table 3:** Incidence of clinical events

Variables, n (%)	Overall	BP-SES	DP-EES	*p* value
	n = 141	n = 44	n = 100	
All cause death	7 (5.0)	2 (4.6)	5 (5.0)	0.906
Cardiac death	6 (4.2)	2 (4.6)	4 (4.0)	0.571
Myocardial infarction	2 (1.4)	2 (4.6)	0 (0)	0.585
Target lesion revascularization	7 (5.0)	5 (11.4)	2 (2.0)	*0.003*
Target vessel revascularization	9 (6.4)	5 (11.4)	4 (4.0)	0.595
Non-target vessel vascularization	7 (5.0)	2 (4.6)	5 (5.0)	0.837
Stent thrombosis	2 (1.4)	2 (4.6)	0 (0.0)	0.448
Major adverse cardiac event	12 (8.5)	6 (13.6)	6 (6.0)	0.878
Device-oriented clinical endpoint	12 (8.5)	6 (13.6)	6 (6.0)	*0.035*

*BP-SES* biodegradable polymer sirolimus-eluting stent, *DP-EES* durable polymer everolimus-eluting stent

### Predictors for TLR

Logistic regression analyses demonstrated that BP-SES use and hemodialysis were significantly associated with higher incidence of TLR (Table 4). Rotational atherectomy tended to be associated with the occurrence of TLR. Univariate and multivariate analyses to identify factors for the incidence of each clinical event are shown in Additional file 1: Tables S5–S13.

**Table 4:** Univariate analysis to identify independent factors for the incidence of TLR

	Univariate		
	Odds ratio	95% CI	*p* value
Hypertension	0.820	0.093–7.225	0.858
Dyslipidemia	0.399	0.073–1.565	0.166
Chronic kidney disease	0.795	0.169–3.745	0.771
Hemodialysis	10.370	2.104–51.108	*0.004*
Smoking	0.730	0.084–6.369	0.776
Previous myocardial infarction	1.668	0.362–7.692	0.512
Previous PCI	0.410	0.079–2.138	0.290
Type B2/C lesion	2.040	0.382–10.906	0.404
Chronic total occlusion	3.813	0.687–21.156	0.126
Rotational atherectomy	5.920	0.998–35.112	0.050
Small stent (≤ 2.5 mm)	0.851	0.185–3.917	0.836
Long stent (≥ 30 mm)	0.861	0.188–3.936	0.847
Multiple stent	1.634	0.357–7.469	0.527
BP-SES usage	6.686	1.234–36.217	*0.028*
ACEI/ARB	0.738	0.136–3.989	0.724
Beta blocker	0.617	0.133–2.872	0.539
Statin	1.128	0.129–9.829	0.913
Insulin	3.455	0.723–16.522	0.120

*ACEI* angiotensin converting enzyme inhibitor, *ARB* angiotensin II receptor blocker, *BP-SES* biodegradable polymer sirolimus-eluting stent, *CI* confidence interval, *PCI* percutaneous coronary intervention, *TLR* target lesion revascularization

### Cost-effectiveness

The BP-SES usage resulted in a cost-increasing of ¥ 209,233.4 per patient within 1 year compared to the DP-EES usage (¥ 3,842,071.14 vs. ¥ 3,632,837.74 per patient). The ICER for the DP-EES usage was ¥ 5,103,253.66 per QALY gained.

## Discussion

The main finding of the present study is that the incidence of TLR and DoCE was significantly greater in patients treated with the BP-SES than in those treated with the DP-EES among patients with diabetes.

### Mechanisms of the greater incidence of TLR in the BP-SES group than in the DP-EES group

In contrast to the CENTURY II randomized trial which included limited number of patients with diabetes and showed the comparable incidence of TLF in the BP-SES and DP-EES [7], the greater incidence of TLR in the BP-SES was demonstrated in the present study. There are several conceivable characteristics of the BP-SES that might affect the higher incidence of TLR than in the DP-EES group, as shown in the present study. First, the sirolimus-releasing biodegradable polymer exclusively exists on the abluminal side of the strut in the BP-SES. The link between struts was not coated by the polymer [6, 20]. Therefore, anti-proliferative properties may be unavailable on the side of vessel wall and lateral side of the strut in BP-SES, in contrast to DP-EES, which has circumferential polymer coating. Second, the greater inflammatory reaction around the strut and subsequent neointimal formation may be accelerated in BP-SES than in DP-EES. Torii et al. investigated the affinity of stent struts for circulating molecules using a swine shunt model [22]. The authors demonstrated that accumulation of monocytes and neutrophils on strut surfaces was greater in the BP-SES than in the DP-EES. The greater inflammatory reaction around the strut of the BP-SES was suggested in a clinical study using optical frequency domain imaging. Sato et al. investigated the incidence of peri-strut low intensity area (PLIA) indicating the presence of accumulated inflammatory cells [23–25]. The authors reported that there was a trend toward higher incidence of PLIA at 1 month after BP-SES implantation than after DP-EES implantation [26]. In addition, Jimba et al. introduced a case of restenosis with inflammatory response eleven months after a BP-SES implantation confirmed by fluorodeoxyglucose with positron emission tomography [27]. The authors reported that increased uptake was observed around the BP-SES although significant fluorodeoxyglucose uptake was not observed around a DP-EES that had been simultaneously implanted in another vessel at the same time of BP-SES implantation. They further reported thickened neointima with a PLIA-like layered pattern and microvascularization within the BP-SES observed by optical coherence tomography. Taken together with these characteristics in BP-SES, the greater inflammatory reaction and subsequent neointimal hyperplasia and/or neoatherosclerosis formation may be accelerated after the implantation of BP-SES than of DP-EES. In fact, the greater in-stent late loss at 9 months in the BP-SES group compared to the DP-EES group was reported in the CENTURY II trial [7].

### Impact of diabetes mellitus on the incidence of TLR after BP-SES implantation

The accelerated inflammatory response around the struts of BP-SES may be further enhanced in patients with diabetes, although the exact pathophysiology for the greater TLR after BP-SES implantation in patients with diabetes is still unclear. Several studies have demonstrated the interaction between the enhanced inflammatory cell infiltration within coronary plaques and subsequent neointimal hyperplasia after PCI in patients with diabetes [28–30]. Thus, the combination of preexisting enhanced inflammation in target plaque and BP-SES implantation in patients with diabetes may further recruit inflammatory cells and cause prolonged inflammation around struts and subsequent neointimal thickening. The higher prevalence of calcification may also cause greater incidence of TLR after BP-SES implantation in patients with diabetes [31, 32]. Several studies have reported that calcified plaque causes the delamination of polymer on the abluminal side of struts [33, 34]. Because the polymer and anti-proliferative drug exclusively exist on the abluminal side in the BP-SES, delamination of the polymer by calcified plaque may cause significant loss of anti-proliferative effect. The attenuated effects of sirolimus under high glucose conditions may also affect the greater incidence of TLR after BP-SES implantation in patients with diabetes [35]. In contrast to a limited percentage of patients with diabetes were included in the CENTURY trial (24%) [6] and the ULISSE registry (29%) [8], the exclusive cohort with diabetes in the present study might be attributed to highlight the higher incidence of TLR after BP-SES implantation than after DP-EES implantation.

### Other devices in patients with diabetes mellitus

In the present study, incidence of TLR in the BP–SES group was higher than that in the DP-EES group. On the other hand, previous studies have not always demonstrated the worse clinical outcomes in the biodegradable polymer-DES compared with the durable-polymer DES among patients with diabetes. Tang et al. reported that the incidence of TLR within 2 years was not significantly different between patients treated with the biodegradable polymer-DES and those treated with the durable-polymer 2nd generation-DES among patients with diabetes [36]. In subgroup analyses of the BIOFLOW trial series, the comparable incidence of TLF within 1 year was demonstrated in patients with Orsiro™ BP-SES (Biotronik, Bülach, Switzerland) and those with the Xience™ DP-EES among patients with diabetes [19]. In addition, the comparable incidence of TLR within 1 year between patients with and without diabetes was demonstrated among patients treated with the Orsiro™ BP-SES in an all-comers registry [37]. These findings suggested that biodegradable-polymer DESs may not always cause higher incidence of TLR than durable-polymer DESs in patients with diabetes. Thus, the results in the present study was not always applicable to other types of BP-SES or biodegradable polymer-DES. Although it is still impossible to give a conclusive comment, the unique characteristics of polymer coating and drug distribution in Ultimaster™ BP-SES might cause the higher incidence of TLR than in the DP-EES group among patients with diabetes in the present study.’. In fact, a recent trial demonstrated the lower incidence of TLR in another type of BP-SES (Orsiro™) than in the polymer free biolimus-eluting stent (BioFreedom™, Biosensors, Morges, Switzerland) [38]. Several new devices may be the future options for patients with diabetes. A recent study demonstrated the feasibility and safety of a Tapered Biodegradable Polymer-Coated Sirolimus-Eluting Stent in long lesion in real-world practices [39]. Because patients with diabetes often have diffuse lesions with small vessel diameter requiring multiple stents [40], a long-tapered DES may contribute to avoiding overlapping stentings. A bioresorbable scaffolds (BRS) may also be an alternative to metallic DES in diabetic patients. A recent study demonstrated the comparable midterm safety and efficacy outcomes of everolimus-eluting BRS in diabetic patients when historically compared with modern DES [41]. Although a concern for the incidence of thrombosis through the process of resorption still exists in the current BRS.

[42], further development of new generation BRS may overcome this problem and become a feasible option for diabetic patients.

### Cost-effectiveness

In Japan, willing to pay has been reported as approximately \ 4,000,000–5,000,000 per QALY [21, 43]. In the present study, the ICER for the DP-EES usage was calculated as \ 5,103,253.66 per QALY gained compared to the BP-SES usage based on 1-year clinical outcomes among patients with diabetes. Because it is marginal, it is difficult to conclude if the usage of DP-EES is cost-effective. Further studies with larger cohorts and longer follow-up duration are required to assess cost-effectiveness.

## Limitations

Several limitations need to be mentioned. First, the present study was a retrospective observational study conducted in a single center. Second, the present study exclusively included patients with stable coronary disease to focus the difference in stent type. Further studies including patients with acute coronary syndrome may yield additional insights into the present topic. Third, potential confounders for the incidence of adverse events may not all have been removed, although the adjustment using multivariate analysis was conducted. Fourth, the duration of clinical follow-up was limited. Fifth, because the present study was not a randomized trial, the selection of stent type was left at the operators' discretion. This might be a potential selection bias.

## Conclusion

BP-SES was associated with the greater incidence of TLR than DP-EES in patients with diabetes. Further studies with larger cohorts and longer follow-up are required to confirm the present results.

## Supplementary information

**Additional file 1:** Supplementary materials

## Data Availability

The datasets generated and analyzed during the current study are not publicly available due to individual privacy but are available from the corresponding author on reasonable request.

## Abbreviations

BMS: Bare metal stents
BP-SES: Biodegradable polymer sirolimus-eluting stent
BRS: Bioresorbable scaffolds
CI: Confidence interval
DES: Drug-eluting stents
DoCE: Device-oriented clinical endpoint
DP-EES: Durable polymer everolimus-eluting stent
ICER: Incremental cost-effectiveness ratio
OR: Odds ratio
PCI: Percutaneous coronary intervention
PLIA: Peri-strut low intensity area
QALY: Quality-adjusted life-year
TLF: Target lesion failure
TLR: Target lesion revascularization
