# Supplementary material for: Clinical outcome of biodegradable polymer sirolimus-eluting stent and durable polymer everolimus-eluting stent in patients with diabetes
Source: Cardiovasc Diabetol. 2020 Oct 1;19:162. doi: 10.1186/s12933-020-01145-x (PMC7531093; doi:10.1186/s12933-020-01145-x)
Supplement: Supplementary file 1 — Additional file 1: Supplementary materials [file 12933_2020_1145_MOESM1_ESM.docx]

**SUPPLEMENTARY MATERIALS**

**Clinical outcome of biodegradable polymer sirolimus-eluting stent and durable polymer everolimus-eluting stent in patients with diabetes**

Ryota Kakizaki*, MD, PhD; Yoshiyasu Minami*, MD, PhD; Masahiro Katamine, MD; Aritomo Katsura, MD; Yusuke Muramatsu, MD, PhD; Takuya Hashimoto, MD, PhD; Kentaro Meguro, MD, PhD; Takao Shimohama, MD, PhD; Junya Ako, MD, PhD

Department of Cardiovascular Medicine, Kitasato University School of Medicine, Sagamihara, Japan

**Supplementary methods**

**Supplementary tables 1-13**

**Supplementary figure 1**

**Supplementary references**

**Supplementary** **methods**

***PCI procedure***

Intracoronary imaging devices were routinely used in all cases unless the devices could not be advanced into the lesion. Physicians in charge of emergency cases tended to choose durable polymer everolimus-eluting stent (DP-EES) at our institute during the study period. Thus, the number of cases with ST-segment elevation myocardial infarction (STEMI) was relatively smaller in the biodegradable polymer sirolimus-eluting stent (BP-SES) group than in the DP-EES group in all PCI cohort of the present study. Before the stent implantation, all patients with acute coronary syndrome (ACS) received aspirin 200 mg and clopidogrel 300 mg or prasugrel 20 mg.

***Follow-up***

Intensive lipid-lowering therapy was encouraged to maintain low-density lipoprotein cholesterol (LDL-C) < 70 mg/dL in patients with ACS and LDL-C < 100 mg/dL in patients with stable angina pectoris (SAP), using statin or other recommended drugs in outpatient clinic. Follow-up angiography was not mandatory.

***Study definitions***

Acute coronary syndrome consisted of ST elevation myocardial infarction (STEMI), non-STEMI (NSTEMI), and unstable angina pectoris. STEMI was defined as continuous chest pain lasting >30 min, arrival at the hospital within 12 h from the onset of chest pain, ST-segment elevation >0.1 mV in ≥2 contiguous leads, or new left bundle-branch block on the 12-lead electrocardiogram (ECG) and elevated cardiac markers (creatine kinase-MB or troponin T/I). NSTEMI was defined as ischemic symptoms with elevated cardiac markers in the absence of ST-segment elevation on ECG. Unstable angina was defined as angina at rest, accelerating angina, or new-onset angina, without the elevation of cardiac markers. Hypertension was defined as arterial blood pressure > 140/90 mm Hg or the use of antihypertensive medication. Dyslipidemia was defined as high-density lipoprotein cholesterol < 40 mg/dL, low-density lipoprotein cholesterol > 140 mg/dL, or triglycerides > 150 mg/dL, or the use of dyslipidemia medication. Diabetes mellitus was defined as symptoms of diabetes plus casual plasma glucose concentration > 200 mg/dL, fasting plasma glucose concentration > 126 mg/dL, 2-h plasma glucose concentration > 200 mg/dL during a 75-g oral glucose tolerance test, or the use of diabetes medication. Chronic kidney disease was defined as estimated glomerular filtration rate of < 60 mL/min per 1.73 m^2^.

Ischemia-driven revascularization was defined as a repeat PCI or bypass surgery of the lesions with either: acute myocardial infarction, unstable angina, SAP, or documented silent ischemia. In patients with SAP or asymptomatic myocardial ischemia, the presence of ischemia was determined by scintigram, physiology (e.g. fractional flow reserve) or the combination of typical angina and angiographical severe stenosis. On the basis of angiographical findings, revascularization was further categorized as target lesion (previous PCI site) revascularization (TLR), and target vessel (previous PCI vessel) revascularization (TVR), and non-TVR (NTVR) (previously untreated segment in any 1 of 3 coronary arteries), whichever occurred first in the patient.

***Propensity score-matching analysis***

Because there might be potential bias in baseline clinical characteristics between the BP-SES group and the DP-EES group, we further compared the incidence of adverse events in an adjusted cohort which was identified using a propensity score-matched analysis. To control selection bias for the incidence of clinical events, the nearest neighbor one-to-two pair matching was performed based on baseline clinical factors which might affect the incidence of outcome measures. Factors included sex, hypertension, dyslipidemia, chronic kidney disease, lesion type, small vessel, long lesion, rotational atherectomy, and medications including thienopyridine, angiotensin converting enzyme inhibitor or angiotensin II receptor blocker, and beta blocker. A caliper width of 0.2 of the standard deviation of the logit of the propensity score was applied for the developed propensity score. We finally identified 34 lesions in 31 patients in the BP-SES group and 68 lesions in 61 patients in the DP-EES group and compared the incidence of adverse events between the adjusted two groups.

***Cost-effectiveness analysis***

Based on previous reports, we calculated the cost of initial procedure as ¥ 2,887,685, following standard care as ¥ 682,183, death as ¥ 1,955,000, target lesion related myocardial infarction with PCI as ¥ 2,416,810, target lesion related myocardial infarction causing death as ¥ 2,831,180, TLR as ¥ 2,386,974 [1], [2], [3], [4], [5], [6], [7]. The average cost within 1 year per patient was calculated by dividing the total cost by the number of patients in each group. We used quality-adjusted life-year (QALY) as an outcome measurement in analysis for incremental cost-effectiveness ratio (ICER) [8]. Based on previous studies, we set quality of life (QOL) for MI and TLR at 0.67 and 0.79, respectively [6], [7].

**Table S1. Time trend in the use of BP-SES and DP-EES**

|  | **Overall** | **BP-SES** | **DP-EES** |  |
| --- | --- | --- | --- | --- |
|  | **n = 165** | **n = 48** | **n = 117** | ***p* value** |
| Oct 2015 – Jul 2016 | 40 (24.2) | 7 (14.6) | 33 (28.2) | 0.179 |
| Aug 2016 – May 2017 | 70 (42.4) | 23 (47.9) | 47 (40.2) |  |
| Jun 2017 – Mar 2018 | 55 (33.3) | 18 (37.5) | 37 (31.6) |  |

BP-SES, biodegradable polymer sirolimus-eluting stent; DP-EES, durable polymer everolimus-eluting stent.**Table S2. Clinical characteristics in an adjusted cohort**

|  | **Overall** | **BP-SES** | **DP-EES** |  |
| --- | --- | --- | --- | --- |
|  | **n = 90** | **n = 31** | **n = 60** | ***p* value** |
| Age, years | 70 (61.9-77) | 72 (58-78) | 70 (64-77) | 0.558 |
| Male, n (%) | 76 (84.4) | 26 (83.9) | 50 (83.3) | 0.948 |
| Body mass index | 22.0 (24.7-26.8) | 24.7 (19.7-26.8) | 24.7 (22.4-26.8) | 0.442 |
| Follow-up duration, days | 334 (382-472) | 364 (321-426) | 386 (340-520) | 0.150 |
| Risk factor, n (%) |  |  |  |  |
| Hypertension | 74 (82.2) | 27 (87.1) | 48 (80.0) | 0.399 |
| Dyslipidemia | 71 (78.9) | 26 (83.9) | 46 (76.7) | 0.423 |
| Chronic kidney disease | 50 (55.6) | 17 (54.8) | 34 (56.7) | 0.868 |
| Hemodialysis | 13 (14.4) | 4 (12.9) | 9 (15.0) | 0.787 |
| Smoking | 16 (17.8) | 8 (25.8) | 8 (13.3) | 0.139 |
| Family history of IHD | 20 (22.5) | 9 (29.0) | 12 (20.3) | 0.354 |
| Previous myocardial infarction | 25 (27.8) | 7 (22.6) | 18 (30.0) | 0.452 |
| Previous PCI | 40 (44.4) | 11 (35.5) | 30 (50.0) | 0.187 |
| Previous CABG | 2 (2.2) | 2 (6.5) | 0(0) | **0.047** |
| Medication at PCI, n (%) |  |  |  |  |
| Aspirin | 81 (90.0) | 28 (90.3) | 53 (88.3) | 0.774 |
| Thienopyridine | 73 (81.1) | 26 (83.9) | 48 (80.0) | 0.653 |
| Statin | 74 (82.2) | 27 (87.1) | 48 (80.0) | 0.399 |
| ACEI/ARB | 66 (73.3) | 22 (71.0) | 45 (75.0) | 0.679 |
| Beta blocker | 56 (62.2) | 19 (61.3) | 38 (63.3) | 0.849 |
| Calcium channel blocker | 44 (48.9) | 15 (48.4) | 29 (48.3) | 0.996 |
| Insulin | 13 (14.6) | 4 (13.3) | 9 (15.0) | 0.832 |
| Laboratory findings |  |  |  |  |
| Triglycerides, mg/dL | 116 (92-176) | 111 (92-152) | 119 (92-200) | 0.315 |
| LDL-C, mg/dL | 87 (67-110) | 89 (66-104) | 86 (68-115) | 0.464 |
| HDL-C, mg/dL | 51 (42-59) | 53 (43-68) | 49 (41-57) | 0.090 |
| HbA1c, % | 6.8 (6.4-7.3) | 6.8 (6.3-7.4) | 6.8 (6.3-7.2) | 0.728 |
| eGFR, mL/min/1.73m^2^ | 53 (38-68) | 58 (39-70) | 52 (32-68) | 0.333 |
| BNP, pg/mL | 91 (42-344) | 92 (34-240) | 99 (42-500) | 0.481 |
| Left ventricular ejection fraction, % | 60 (54-66) | 60 (54-68) | 60 (53-66) | 0.677 |

ACEI, angiotensin converting enzyme inhibitor; ARB, angiotensin II receptor blocker; BNP, brain natriuretic peptide; BP-SES, biodegradable polymer sirolimus-eluting stent; CABG, coronary artery bypass graft; DP-EES durable polymer everolimus-eluting stent; eGFR, estimated glomerular filtration rate; HbA1c, hemoglobin A1c; HDL-C, high density lipoprotein cholesterol; IHD, ischemic heart disease; LDL-C, low-density lipoprotein cholesterol; PCI, percutaneous coronary intervention.

**Table S3. Lesion and procedural characteristics in an adjusted cohort**

|  | **Overall** | **BP-SES** | **DP-EES** |  |
| --- | --- | --- | --- | --- |
|  | **n = 102** | **n = 34** | **n = 68** | ***p* value** |
| Target vessel, n (%) |  |  |  | 0.104 |
| Left main trunk | 4 (3.) | 1 (2.9) | 3 (4.4) |  |
| Left anterior descending artery | 52 (51.0) | 15 (44.1) | 37 (54.4) |  |
| Left circumflex artery | 10 (9.8) | 1 (2.9) | 9 (13.2) |  |
| Right coronary artery | 36 (35.2) | 17 (50.0) | 19 (27.9) |  |
| Type B2/C lesion, n (%) | 63 (61.8) | 21 (61.8) | 42 (61.8) | 1.000 |
| QCA pre PCI |  |  |  |  |
| Lesion length, mm | 23.9 (13.4-36.4) | 25.7 (12.5-39.7) | 23.2 (14.1-35.6) | 0.840 |
| Reference vessel diameter, mm | 2.49 (2.12-3.03) | 2.38 (2.04-2.87) | 2.53 (2.18-3.12) | 0.319 |
| Minimum lumen diameter, mm | 0.90 (0.44-1.22) | 0.84 (0.57-1.08) | 0.92 (0.39-1.25) | 0.972 |
| Diameter stenosis, % | 65 (49-80) | 65 (57-78) | 65 (48-81) | 0.994 |
| QCA post PCI |  |  |  |  |
| Reference vessel diameter, mm | 2.77 (2.46-3.16) | 2.82 (2.48-3.06) | 2.73 (2.42-3.19) | 0.975 |
| Minimum lumen diameter, mm | 2.43 (2.05-2.74) | 2.43 (2.13-2.75) | 2.43 (2.05-2.71) | 0.801 |
| Diameter stenosis, % | 12 (6-19) | 11 (5-19) | 12 (6-20) | 0.662 |
| Rotational atherectomy, n (%) | 6 (5.9) | 2 (5.9) | 4 (5.9) | 1.000 |
| Bifurcation, n (%) | 25 (24.5) | 9 (26.5) | 16 (23.5) | 0.745 |
| Chronic total occlusion, n (%) | 11 (10.8) | 3 (8.8) | 8 (11.8) | 0.652 |
| Imaging device, n (%) | 102 (100) | 34 (100) | 68 (100) | - |
| Number of stents, n | 2 (1—2) | 2 (1-2) | 1 (1-2) | 0.152 |
| Total stent length, mm | 38 (28-61) | 38 (28-62) | 38 (23-56) | 0.231 |
| Minimum stent diameter, mm | 2.75 (2.5-3.0) | 2.5 (2.5-3.25) | 2.75 (2.5-3.25) | 0.303 |

BP-SES, biodegradable polymer sirolimus-eluting stent; DP-EES durable polymer everolimus-eluting stent; PCI, percutaneous coronary intervention; QCA, quantitative coronary angiography.

**Table S4. Incidence of clinical events in an adjusted cohort**

|  | **Overall** | **BP-SES** | **DP-EES** | ***p* value** |
| --- | --- | --- | --- | --- |
| Variables, n (%) | **n = 90** | **n = 31** | **n = 60** |  |
| All cause death | 5 (5.6) | 2 (6.5) | 3 (5.0) | 0.773 |
| Cardiac death | 4 (4.4) | 2 (6.5) | 2 (3.3) | 0.517 |
| Myocardial infarction | 1 (1.1) | 1 (3.2) | 0 (0) | 0.874 |
| Target lesion revascularization | 4 (4.4) | 4 (12.9) | 0 (0) | **0.001** |
| Target vessel revascularization | 5 (5.6) | 4 (12.9) | 1 (1.7) | 0.875 |
| Non-target vessel vascularization | 5 (5.6) | 2 (6.5) | 3 (5.0) | 0.326 |
| Stent thrombosis | 1 (1.1) | 1 (3.2) | 0 (0) | **0.020** |
| Major adverse cardiac event | 7 (7.8) | 5 (16.1) | 2 (3.3) | 0.468 |
| Device-oriented clinical endpoint | 7 (7.8) | 5 (16.1) | 2 (3.3) | **0.001** |

BP-SES, biodegradable polymer sirolimus-eluting stent; DP-EES durable polymer everolimus-eluting stent; p value was estimated in Log-rank test for all cause death and Fine-Gray's test for other events.

**Table S5. Univariate and multivariate analysis to identify independent factors for the incidence of TLR**

|  | **Univariate** | | | **Multivariate** | | |
| --- | --- | --- | --- | --- | --- | --- |
|  | **Odds ratio** | **95% CI** | ***p* value** | **Odds ratio** | **95% CI** | ***p* value** |
| Hypertension | 0.820 | 0.093-7.225 | 0.858 |  |  |  |
| Dyslipidemia | 0.399 | 0.073-1.565 | 0.166 |  |  |  |
| Chronic kidney disease | 0.795 | 0.169-3.745 | 0.771 |  |  |  |
| Hemodialysis | 10.370 | 2.104-51.108 | **0.004** | 13.627 | 2.350-79.005 | **0.004** |
| Smoking | 0.730 | 0.084-6.369 | 0.776 |  |  |  |
| Previous myocardial infarction | 1.668 | 0.362-7.692 | 0.512 |  |  |  |
| Previous PCI | 0.410 | 0.079-2.138 | 0.290 |  |  |  |
| Type B2/C lesion | 2.040 | 0.382-10.906 | 0.404 |  |  |  |
| Chronic total occlusion | 3.813 | 0.687-21.156 | 0.126 |  |  |  |
| Rotational atherectomy | 5.920 | 0.998-35.112 | 0.050 |  |  |  |
| Small stent (≤2.5 mm) | 0.851 | 0.185-3.917 | 0.836 |  |  |  |
| Long stent (≥30 mm) | 0.861 | 0.188-3.936 | 0.847 |  |  |  |
| Multiple stent | 1.634 | 0.357-7.469 | 0.527 |  |  |  |
| BP-SES usage | 6.686 | 1.234-36.217 | **0.028** | 8.823 | 1.328-58.597 | **0.024** |
| ACEI/ARB | 0.738 | 0.136-3.989 | 0.724 |  |  |  |
| Beta blocker | 0.617 | 0.133-2.872 | 0.539 |  |  |  |
| Statin | 1.128 | 0.129-9.829 | 0.913 |  |  |  |
| Insulin | 3.455 | 0.723-16.522 | 0.120 |  |  |  |

ACEI, angiotensin converting enzyme inhibitor; ARB, angiotensin II receptor blocker; BP-SES, biodegradable polymer sirolimus-eluting stent; CI, confidence interval; PCI, percutaneous coronary intervention; TLR, target lesion revascularization.

**Table S6. Univariate and multivariate analysis to identify independent factors for the incidence of all death**

|  | **Univariate** | | | **Multivariate** | | |
| --- | --- | --- | --- | --- | --- | --- |
|  | **Odds ratio** | **95% CI** | ***p* value** | **Odds ratio** | **95% CI** | ***p* value** |
| Hypertension | 0.964 | 0.104-8.947 | 0.974 |  |  |  |
| Dyslipidemia | 0.246 | 0.057-1.054 | 0.059 |  |  |  |
| Chronic kidney disease | 4.448 | 0.493-40.108 | 0.183 |  |  |  |
| Hemodialysis | 13.725 | 2.099-89.743 | **0.006** | 12.272 | 1.899-79.297 | **0.008** |
| Smoking | 2.844 | 0.671-12.052 | 0.156 |  |  |  |
| Previous PCI | 0.623 | 0.217-1.792 | 0.380 |  |  |  |
| Type B2/C lesion | 1.341 | 0.464-3.874 | 0.588 |  |  |  |
| Chronic total occlusion | 3.156 | 0.671-14.833 | 0.146 |  |  |  |
| Small stent (≤2.5 mm) | 3.634 | 0.625-21.130 | 0.151 |  |  |  |
| Long stent (≥30 mm) | 4.817 | 0.537-43.215 | 0.160 |  |  |  |
| Multiple stent | 3.826 | 0.858-17.061 | 0.079 | 3.098 | 0.730-13.139 | 0.125 |
| BP-SES usage | 0.804 | 0.137-4.719 | 0.809 |  |  |  |
| ACEI/ARB | 2.158 | 0.237-19.622 | 0.495 |  |  |  |
| Beta blocker | 0.454 | 0.108-1.909 | 0.281 |  |  |  |
| Statin | 0.541 | 0.090-3.249 | 0.502 |  |  |  |
| Insulin | 1.460 | 0.319-6.689 | 0.626 |  |  |  |

ACEI, angiotensin converting enzyme inhibitor; ARB, angiotensin II receptor blocker; BP-SES, biodegradable polymer sirolimus-eluting stent; CI, confidence interval; PCI, percutaneous coronary intervention.

**Table S7. Univariate and multivariate analysis to identify independent factors for the incidence of cardiac death**

|  | **Univariate** | | | **Multivariate** | | |
| --- | --- | --- | --- | --- | --- | --- |
|  | **Odds ratio** | **95% CI** | ***p* value** | **Odds ratio** | **95% CI** | ***p* value** |
| Hypertension | 0.820 | 0.089-7.583 | 0.861 |  |  |  |
| Dyslipidemia | 0.339 | 0.060-1.899 | 0.218 |  |  |  |
| Chronic kidney disease | 3.773 | 0.421-33.849 | 0.236 |  |  |  |
| Hemodialysis | 10.370 | 1.693-63.532 | **0.011** | 9.450 | 1.442-61.909 | **0.019** |
| Smoking | 1.829 | 0.299-11.193 | 0.514 |  |  |  |
| Previous PCI | 0.789 | 0.212-2.931 | 0.723 |  |  |  |
| Type B2/C lesion | 1.061 | 0.285-3.952 | 0.930 |  |  |  |
| Chronic total occlusion | 3.813 | 0.874-16.642 | 0.075 | 2.911 | 0.555-15.268 | 0.206 |
| Small stent (≤2.5 mm) | 2.986 | 0.516-17.296 | 0.222 |  |  |  |
| Long stent (≥30 mm) | 4.085 | 0.458-36.456 | 0.208 |  |  |  |
| Multiple stent | 3.143 | 0.764-12.921 | 0.112 |  |  |  |
| BP-SES usage | 0.974 | 0166-5.705 | 0.977 |  |  |  |
| ACEI/ARB | 1.835 | 0.203-16.605 | 0.589 |  |  |  |
| Beta blocker | 0.617 | 0.112-3.390 | 0.579 |  |  |  |
| Statin | 0.448 | 0.075-2.685 | 0.379 |  |  |  |
| Insulin | 1.766 | 0.416-7.496 | 0.441 |  |  |  |

ACEI, angiotensin converting enzyme inhibitor; ARB, angiotensin II receptor blocker; BP-SES, biodegradable polymer sirolimus-eluting stent; CI, confidence interval; PCI, percutaneous coronary intervention.

**Table S8. Univariate analysis to identify independent factors for the incidence of myocardial infarction**

|  | **Univariate** | | |
| --- | --- | --- | --- |
|  | **Odds ratio** | **95% CI** | ***p* value** |
| Chronic kidney disease | 0.598 | 0.036-9.922 | 0.720 |
| Hemodialysis | 6.762 | 0.407-112.397 | 0.183 |
| Smoking | 4.552 | 0.277-74.907 | 0.289 |
| Previous myocardial infarction | 2.196 | 0.138-35.049 | 0.578 |
| Previous PCI | 1.063 | 0.067-16.959 | 0.965 |
| Type B2/C lesion | 0.791 | 0.048-13.117 | 0.870 |
| Small stent (≤2.5 mm) | 1.145 | 0.071-18.366 | 0.924 |
| Long stent (≥30 mm) | 0.646 | 0.041-10.310 | 0.758 |
| Multiple stent | 1.203 | 0.075-19.259 | 0.896 |
| Beta blocker | 0.468 | 0.029-7.578 | 0.593 |

CI, confidence interval; PCI, percutaneous coronary intervention.

**Table S9. Univariate and multivariate analysis to identify independent factors for the incidence of TVR**

|  | **Univariate** | | | **Multivariate** | | |
| --- | --- | --- | --- | --- | --- | --- |
|  | **Odds ratio** | **95% CI** | ***p* value** | **Odds ratio** | **95% CI** | ***p* value** |
| Hypertension | 1.109 | 0.131-9.391 | 0.924 |  |  |  |
| Dyslipidemia | 0.516 | 0.125-2.125 | 0.360 |  |  |  |
| Chronic kidney disease | 0.740 | 0.191-2.867 | 0.663 |  |  |  |
| Hemodialysis | 6.133 | 1.482-25.375 | **0.012** | 6.718 | 1.553-29.060 | **0.011** |
| Smoking | 0.539 | 0.064-4.515 | 0.569 |  |  |  |
| Previous myocardial infarction | 1.800 | 0.474-6.837 | 0.388 |  |  |  |
| Previous PCI | 0.842 | 0.225-3.147 | 0.798 |  |  |  |
| Type B2/C lesion | 1.628 | 0.393-6.745 | 0.502 |  |  |  |
| Chronic total occlusion | 2.686 | 0.519-13.902 | 0.239 |  |  |  |
| Rotational atherectomy | 4.171 | 0.752-23.153 | 0.102 |  |  |  |
| Small stent (≤2.5 mm) | 0.910 | 0.240-3.448 | 0.889 |  |  |  |
| Long stent (≥30 mm) | 0.803 | 0.210-3.072 | 0.748 |  |  |  |
| Multiple stent | 1.536 | 0.401-5.879 | 0.531 |  |  |  |
| BP-SES usage | 3.285 | 0.840-12.848 | 0.087 |  |  |  |
| ACEI/ARB | 1.050 | 0.208-5.289 | 0.953 |  |  |  |
| Beta blocker | 0.943 | 0.227-3.914 | 0.93 |  |  |  |
| Statin | 0.636 | 0.123-3.299 | 0.590 |  |  |  |
| Insulin | 3.793 | 0.941-15.285 | 0.061 | 4.259 | 0.988-18.364 | 0.052 |

ACEI, angiotensin converting enzyme inhibitor; ARB, angiotensin II receptor blocker; BP-SES, biodegradable polymer sirolimus-eluting stent; CI, confidence interval; PCI, percutaneous coronary intervention; TVR, target vessel revascularization.

**Table S10. Univariate analysis to identify independent factors for the incidence of NTVR**

|  | **Univariate** | | |
| --- | --- | --- | --- |
|  | **Odds ratio** | **95% CI** | ***p* value** |
| Hypertension | 0.321 | 0.063-1.653 | 0.174 |
| Dyslipidemia | 0.339 | 0.077-1.497 | 0.153 |
| Chronic kidney disease | 3.773 | 0.444-32.082 | 0.224 |
| Hemodialysis | 2.760 | 0.531-14.357 | 0.228 |
| Smoking | 0.730 | 0.090-5.928 | 0.768 |
| Previous myocardial infarction | 1.668 | 0.375-7.417 | 0.501 |
| Previous PCI | 0.789 | 0.171-3.640 | 0.761 |
| Type B2/C lesion | 2.040 | 0.383-10.871 | 0.404 |
| Chronic total occlusion | 3.813 | 0.688-21.138 | 0.126 |
| Small stent (≤2.5 mm) | 1.553 | 0.345-6.981 | 0.566 |
| Long stent (≥30 mm) | 1.658 | 0.319-8.606 | 0.547 |
| Multiple stent | 3.143 | 0.601-16.426 | 0.175 |
| BP-SES usage | 0.974 | 0.177-5.363 | 0.976 |
| ACEI/ARB | 0.379 | 0.084-1.719 | 0.209 |
| Beta blocker | 1.192 | 0.231-6.144 | 0.834 |
| Insulin | 0.706 | 0.080-6.213 | 0.753 |

ACEI, angiotensin converting enzyme inhibitor; ARB, angiotensin II receptor blocker; BP-SES, biodegradable polymer sirolimus-eluting stent; CI, confidence interval; PCI, percutaneous coronary intervention, NTVR, non-target vessel revascularization.

**Table S11. Univariate analysis to identify independent factors for the incidence of ST**

|  | **Univariate** | | |
| --- | --- | --- | --- |
|  | **Odds ratio** | **95% CI** | ***p* value** |
| Chronic kidney disease | 0.598 | 0.036-9.922 | 0.720 |
| Hemodialysis | 6.762 | 0.407-112.397 | 0.183 |
| Smoking | 4.552 | 0.277-74.907 | 0.289 |
| Previous myocardial infarction | 2.196 | 0.138-35.049 | 0.578 |
| Previous PCI | 1.063 | 0.067-16.959 | 0.965 |
| Type B2/C lesion | 0.791 | 0.048-13.117 | 0.870 |
| Small stent (≤2.5 mm) | 1.145 | 0.071-18.366 | 0.924 |
| Long stent (≥30 mm) | 0.646 | 0.041-10.310 | 0.758 |
| Multiple stent | 1.203 | 0.075-19.259 | 0.896 |
| Beta blocker | 0.468 | 0.029-7.578 | 0.593 |

CI, confidence interval; PCI, percutaneous coronary intervention; ST, stent thrombosis.

**Table S12. Univariate and multivariate analysis to identify independent factors for the incidence of MACE**

|  | **Univariate** | | | **Multivariate** | | |
| --- | --- | --- | --- | --- | --- | --- |
|  | **Odds ratio** | **95% CI** | ***p* value** | **Odds ratio** | **95% CI** | ***p* value** |
| Hypertension | 0.739 | 0.147-3.704 | 0.713 |  |  |  |
| Dyslipidemia | 0.393 | 0.113-1.364 | 0.141 |  |  |  |
| Chronic kidney disease | 1.388 | 0.387-4.984 | 0.615 |  |  |  |
| Hemodialysis | 10.656 | 2.913-38.975 | **<0.001** | 9.999 | 2.616-38.210 | **0.001** |
| Smoking | 0.792 | 0.159-3.955 | 0.776 |  |  |  |
| Previous myocardial infarction | 0.631 | 0.163-2.440 | 0.504 |  |  |  |
| Previous PCI | 0.642 | 0.220-1.873 | 0.417 |  |  |  |
| Type B2/C lesion | 1.870 | 0.594-5.888 | 0.285 |  |  |  |
| Chronic total occlusion | 4.752 | 1.421-15.892 | **0.011** | 4.153 | 1.065-16.200 | **0.040** |
| Rotational atherectomy | 2.582 | 0.482-13.822 | 0.268 |  |  |  |
| Small stent (≤2.5 mm) | 1.367 | 0.414-4.516 | 0.608 |  |  |  |
| Long stent (≥30 mm) | 1.508 | 0.434-5.245 | 0.518 |  |  |  |
| Multiple stent | 2.030 | 0.702-5.868 | 0.191 |  |  |  |
| BP-SES usage | 2.245 | 0.659-7.652 | 0.196 |  |  |  |
| ACEI/ARB | 0.997 | 0.251-3.966 | 0.997 |  |  |  |
| Beta blocker | 0.738 | 0.215-2.541 | 0.631 |  |  |  |
| Statin | 0.594 | 0.146-2.413 | 0.467 |  |  |  |
| Insulin | 2.041 | 0.631-6.607 | 0.234 |  |  |  |

ACEI, angiotensin converting enzyme inhibitor; ARB, angiotensin II receptor blocker; BP-SES, biodegradable polymer sirolimus-eluting stent; CI, confidence interval; MACE, major adverse cardiovascular events, PCI, percutaneous coronary intervention.

**Table S13. Univariate and multivariate analysis to identify independent factors for the incidence of DoCE**

|  | **Univariate** | | | **Multivariate** | | |
| --- | --- | --- | --- | --- | --- | --- |
|  | **Odds ratio** | **95% CI** | ***p* value** | **Odds ratio** | **95% CI** | ***p* value** |
| Hypertension | 0.739 | 0.147-3.704 | 0.713 |  |  |  |
| Dyslipidemia | 0.393 | 0.113-1.364 | 0.141 |  |  |  |
| Chronic kidney disease | 1.388 | 0.387-4.984 | 0.615 |  |  |  |
| Hemodialysis | 10.656 | 2.913-38.975 | **<0.001** | 9.999 | 2.616-38.210 | **0.001** |
| Smoking | 0.792 | 0.159-3.955 | 0.776 |  |  |  |
| Previous myocardial infarction | 0.631 | 0.163-2.440 | 0.504 |  |  |  |
| Previous PCI | 0.642 | 0.220-1.873 | 0.417 |  |  |  |
| Type B2/C lesion | 1.870 | 0.594-5.888 | 0.285 |  |  |  |
| Chronic total occlusion | 4.752 | 1.421-15.892 | **0.011** | 4.153 | 1.065-16.200 | **0.040** |
| Rotational atherectomy | 2.582 | 0.482-13.822 | 0.268 |  |  |  |
| Small stent (≤2.5 mm) | 1.367 | 0.414-4.516 | 0.608 |  |  |  |
| Long stent (≥30 mm) | 1.508 | 0.434-5.245 | 0.518 |  |  |  |
| Multiple stent | 2.030 | 0.702-5.868 | 0.191 |  |  |  |
| BP-SES usage | 2.245 | 0.659-7.652 | 0.196 |  |  |  |
| ACEI/ARB | 0.997 | 0.251-3.966 | 0.997 |  |  |  |
| Beta blocker | 0.738 | 0.215-2.541 | 0.631 |  |  |  |
| Statin | 0.594 | 0.146-2.413 | 0.467 |  |  |  |
| Insulin | 2.041 | 0.631-6.607 | 0.234 |  |  |  |

ACEI, angiotensin converting enzyme inhibitor; ARB, angiotensin II receptor blocker; BP-SES, biodegradable polymer sirolimus-eluting stent; CI, confidence interval; DoCE, device-oriented clinical endpoints, PCI, percutaneous coronary intervention.

**Figure S1. Simple decision tree representing costs and outcomes of BP-SES and DP-EES**

**
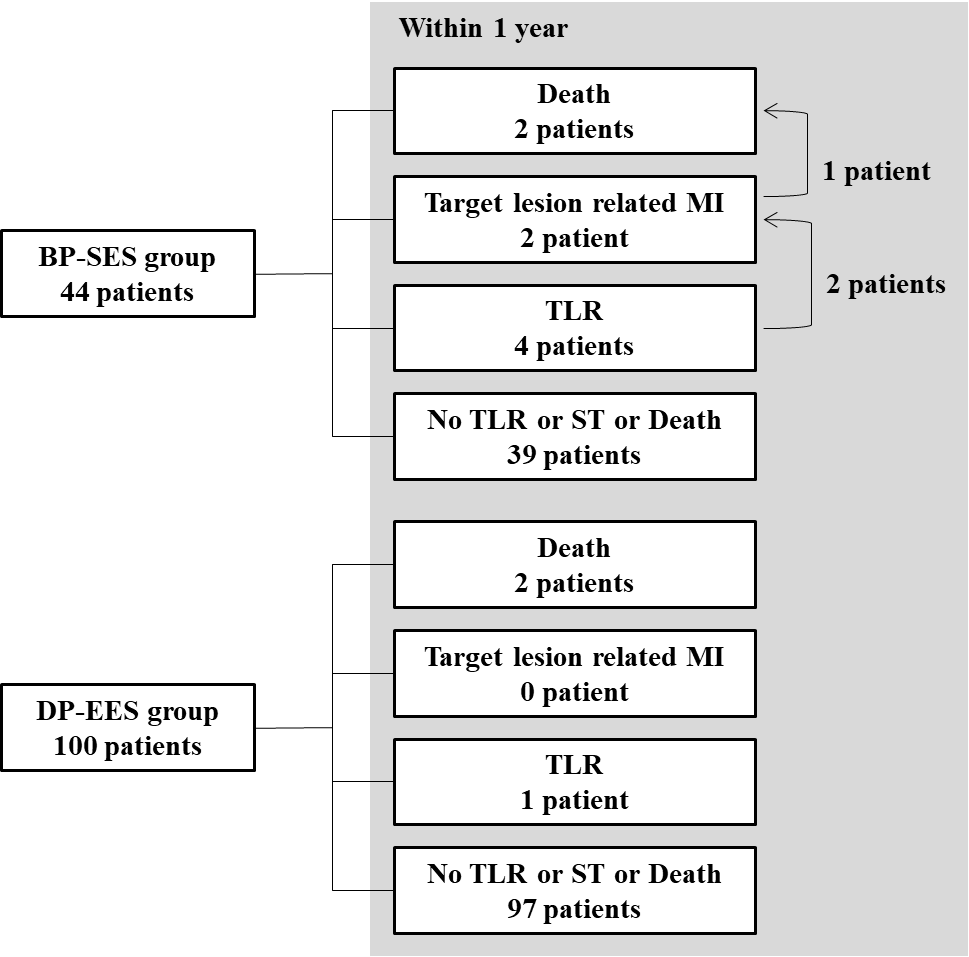
**

**References**

[1] Tanihata S, Nishigaki K, Kawasaki M, Takemura G, Minatoguchi S, Fujiwara H. Outcomes of patients with stable low-risk coronary artery disease receiving medical- and PCI-preceding therapies in Japan - J-SAP study 1-1. Circ J 2006;70:365–9. https://doi.org/10.1253/circj.70.365.

[2] Evans E, Imanaka Y, Sekimoto M, Ishizaki T, Hayashida K, Fukuda H, et al. Risk adjusted resource utilization for AMI patients treated in Japanese hospitals. Health Econ 2007;16:347–59. https://doi.org/10.1002/hec.1177.

[3] Sugimoto K, Kobayashi Y, Kuroda N, Komuro I. Cost analysis of sirolimus-eluting stents in the Japanese health insurance system. Int Heart J 2009;50:723–30. https://doi.org/10.1536/ihj.50.723.

[4] Shimizu T, Ohno T, Ando J, Fujita H, Nagai R, Motomura N, et al. Mid-term results and costs of coronary artery bypass vs drug-eluting stents for unprotected left main coronary artery disease. Circ J 2010;74:449–55. https://doi.org/10.1253/circj.CJ-09-0586.

[5] Pietzsch JB, Geisler BP, Ikeno F. Cost-effectiveness of orbital atherectomy compared to rotational atherectomy in treating patients with severely calcified coronary artery lesions in Japan. Cardiovasc Interv Ther 2018;33:328–36. https://doi.org/10.1007/s12928-017-0488-3.

[6] Takura T, Tachibana K, Isshiki T, Sumitsuji S, Kuroda T, Mizote I, et al. Preliminary report on a cost-utility analysis of revascularization by percutaneous coronary intervention for ischemic heart disease. Cardiovasc Interv Ther 2017;32:127–36. https://doi.org/10.1007/s12928-016-0401-5.

[7] Kodera S, Morita H, Kiyosue A, Ando J, Komuro I. Cost-effectiveness of percutaneous coronary intervention compared with medical therapy for ischemic heart disease in Japan. Circ J 2019;83:1498–505. https://doi.org/10.1253/circj.CJ-19-0148.

[8] Kodera S, Kiyosue A, Ando J, Akazawa H, Morita H, Watanabe M, et al. Cost-effectiveness analysis of cardiovascular disease treatment in Japan. Int Heart J 2017;58:847–52. https://doi.org/10.1536/ihj.17-365.
